# Modeling reproductive and pregnancy-associated tissues using organ-on-chip platforms: challenges, limitations, and the high throughput data frontier

**DOI:** 10.3389/fbioe.2025.1568389

**Published:** 2025-04-01

**Authors:** Nina Truong, Abir Zahra, Ryan C. V. Lintao, Rahul Chauhan, Giovana Fernanda Bento, Manuel Vidal Jr., Sungjin Kim, Po Yi Lam, Thomas Conrads, Kelly Conrads, Arum Han, Ramkumar Menon, Lauren S. Richardson

**Affiliations:** ^1^ John Sealy School of Medicine, University Blvd., Galveston, TX, United States; ^2^ Institute of Reproductive Health, National Institutes of Health, University of the Philippines Manila, Manila, Philippines; ^3^ Division of Basic Science and Translational Research, Department of Obstetrics and Gynecology, The University of Texas Medical Branch at Galveston, Galveston, TX, United States; ^4^ Department of Pathology, Botucatu Medical School, São Paulo State University, São Paulo, Brazil; ^5^ College of Medicine, San Beda University, Manila, Philippines; ^6^ Department of Chemistry, College of Science, De La Salle University Manila, Manila, Philippines; ^7^ Department of Biomedical Engineering and Electrical and Computer Engineering, Texas A&M University, College Station, TX, United States; ^8^ Gynecologic Cancer Center of Excellence, Gynecologic Surgery and Obstetrics, Uniformed Services University of the Health Sciences, Bethesda, MD, United States; ^9^ Women’s Health Integrated Research Center, Women’s Service Line, Inova Health System, Falls Church, VA, United States

**Keywords:** organ-on-chip, pregnancy, reproduction, *in-vitro* tools, cell culture

## Abstract

Over the past decade, organ-on-chip technology (microphysiological systems or tissue chips) has reshaped *in-vitro* physiological and pathological modeling and pharmaceutical drug assessment. FDA Modernization Act 2.0 allows for alternatives to animal testing or the use of appropriate non-animal models/new approach methods (NAMs), such as Organ-on-chips (OC) platforms or *in silico* simulation models, to generate pre-clinical *in-vitro* drug trial data for regulatory purposes primes the microfluidic field to have exponential growth in the coming years. The changes in the approaches of regulatory agencies could significantly impact the development of therapeutics for use during pregnancy. However, limitations of the devices and molecular and biochemical assay shortfalls hinder the progress of the OOC field. This review describes available reproductive and pregnancy-related OOC platforms, and the current methodologies utilized to generate endpoint datasets (e.g., microscopic imaging, immunocytochemistry, real-time polymerase chain reaction, cytokine multiplex analysis). Microfluidic platform limitations, such as fewer number of cells or low supernatant volumes and restrictions regarding fabrication materials, are described. Novel approaches (e.g., spatial transcriptomics, imaging cytometry by time of flight, exosomes analysis using Exoview) to overcome these challenges are described. OOC platforms are primed to provide biologically relevant and clinically translational data that can revolutionize *in-vitro* physiological modeling, drug discovery, and toxicologic risk assessment. However, engineering adaptations to increase the throughput of devices (i.e., device arrays) and biological advancements to improve data throughput are both needed for these platforms to reach their full potential.

## Introduction

Organ-on-chips (OOC) or tissue chips are microphysiological systems that mimic human organ architecture and physiology. Functionality OOCs can be utilized to better understand the development of a disease and disease progression and analyze adverse drug effects in organs of interest. Various OOCs have been constructed to model single human organs such as the brain (van der Helm et al., 2016a; Sances et al., 2018; Jeong et al., 2018), liver (Materne et al., 2015; Maschmeyer et al., 2015; Gori et al., 2016; Deng et al., 2019), kidney (An et al., 2015; Paoli and Samitier, 2016; Wilmer et al., 2016; Ashammakhi et al., 2018), lung (Huh, 2015; Konar et al., 2016; Shrestha et al., 2020), and heart (Zhang et al., 2015; Jastrzebska et al., 2016; Ribas et al., 2016), as well as multi-organ systems (Materne et al., 2015; Maschmeyer et al., 2015; Abaci and Shuler, 2015; Jodat et al., 2018; Baert et al., 2020) such as the reproductive systems (Xiao et al., 2017). These 3D systems allow for environmental manipulations such as cell patterning, flow rate, concentration gradients, culture conditions, and organ-organ interactions allowing investigators to control experimental parameters to perform reliable, and reproducible humanized research (Ma et al., 2021). For decades, animal models have been used as the gold standard for *in-vivo* experiments, but due to species differences (anatomical and functional differences between animals and humans) (Ruberte et al., 2023; Dolenšek et al., 2015; Sheng et al., 2010; Soncin et al., 2015; Soncin et al., 2018), financial costs, and ethical implications, animal models remain far from the ideal (Han, 2023). *Ex-vivo* tissue explant models retain human context and biological complexity though they have a short half-life (∼48 h), are difficult to dissect without inducing damage, and must be collected fast to ensure adequate viability (Hughes et al., 2021; Grivel and Margolis, 2009). While *in-vitro* 2-D cell cultures can provide informative data, they lack the cellular context and ability to simulate human organs’ physiological complexity and interactions with their surroundings (Wu et al., 2020). However, the advanced technology of microfluidic OOC systems can help to overcome these limitations and bridge the gap between cell cultures, animal models, and human trials to advance research (Wu et al., 2020).

Within the vast arena of OOC devices, a small portion of platforms exist in the field of women’s health and reproduction. Reproduction is a fundamental life process crucial to the continuation and diversification of all species. The specialized tissues, organs, and hormones of the reproductive system work in concert to enable fertilization and support the ensuing process of embryonic and fetal development. Many aspects of human reproduction are being explored and imitated by OOC devices, such as the female and male reproductive tract (Xiao et al., 2017; Tantengco et al., 2022a), vaginal microbiome (Mahajan et al., 2022), cervix (Yu et al., 2022; Tantengco et al., 2021; Lee M. et al., 2021; Takeuchi et al., 2019; Tantengco et al., 2022b), ovary (Nagashima et al., 2018; Aziz et al., 2017; Saha et al., 2021; Aziz et al., 2020; Lee JH. et al., 2021), testis (Baert et al., 2020), and endometrium (Park et al., 2020; Gnecco et al., 2019; Gnecco et al., 2017; De Bem et al., 2021; Ahn et al., 2021; Bruner-Tran et al., 2017; Govindasamy et al., 2023). Like many other OOC devices, alteration and combinations of these organ structures allow for a wide variety of microenvironments and complex systems to be created. Under different experimental conditions/treatments, numerous properties of these organs/organ systems may be explored, including implantation (Park et al., 2022), 28-day menstrual cycle (Xiao et al., 2017), spermatogenesis, sperm mobility (Yu et al., 2022; Lee M. et al., 2021), and pathologic processes such as cancer development and metastasis (Saha et al., 2021). Furthermore, these devices have been shown to have utility in assessing reproductive organs’ responses to therapeutics (Saha et al., 2021; Shojaei et al., 2021) or toxicant exposure (Aziz et al., 2020).

A subset of these devices has been reported to model pregnancy-related interfaces by recapitulating several tissue layers, organs, and anatomical systems. A few of these OOC models include the placenta (Shojaei et al., 2021; Lee et al., 2016; Mosavati et al., 2020; Pemathilaka et al., 2019; Yin et al., 2019; Blundell et al., 2018; Abostait et al., 2022a; Mandt et al., 2018; Zhu et al., 2018; Kreuder et al., 2020; Boos et al., 2021; Jeong et al., 2024; Vidal et al., 2024; Abostait et al., 2022b; Richardson et al., 2022; Miura et al., 2015; Rabussier et al., 2023a; Mosavati et al., 2022; Ghorbanpour et al., 2023; Blundell et al., 2016)^,^ amnion (Zhu et al., 2020; Richardson et al., 2019a), cervix (Yu et al., 2022; Tantengco et al., 2021; Lee M. et al., 2021; Takeuchi et al., 2019; Tantengco et al., 2022b; Dadkhah et al., 2023), endometrium (Park et al., 2020; Gnecco et al., 2019; Gnecco et al., 2017; De Bem et al., 2021; Ahn et al., 2021; Bruner-Tran et al., 2017), decidua (Rogers et al., 2018; Richardson et al., 2023a; Lintao et al., 2024), vagina (Mahajan et al., 2022; Czechowicz et al., 2022; Tantengco et al., 2022c; Tantengco et al., 2022d), fetal membrane (Park et al., 2022; Richardson L. et al., 2020; Richardson et al., 2020b; Kim et al., 2022), and a combination of these to form organ systems (i.e., vagina-cervix-decidua (Tantengco et al., 2022c; Tantengco OR. et al., 2022), uterine-endometrium-ovary (Park et al., 2020), placenta-decidua-fetal membrane (Kammala et al., 2023; Safarzadeh et al., 2024)). Besides some differences in the process of fabrication and design of the placental-environment OOCs, multiple studies have aimed to investigate the aspects that are related to this organ during pregnancy in different conditions, such as diseases and tests of drug transportation (Mosavati et al., 2022; Ghorbanpour et al., 2023; Pemathilaka et al., 2022; Ko et al., 2022; Cherubini et al., 2023). These microphysiological systems provide countless avenues for innovative research by providing platforms to study 1) the development, characterization, and remodeling of tissues during pregnancy (e.g., sperm locomotion (Yu et al., 2022), trophoblast invasion during implantation (Park et al., 2022), amnion membrane remodeling during gestation (Zhu et al., 2020; Richardson et al., 2019b)); 2) contribute to a better understanding of the physiologic crosstalk between different pregnancy-related feto-maternal organs by providing cellular level resolution of interactions (Park et al., 2020; Zhu et al., 2020; Richardson L. et al., 2020); 3) providing human-based, ethical experimentation models for drug transport across the feto-maternal interfaces (Pemathilaka et al., 2019; Blundell et al., 2018; Richardson et al., 2022; Kammala et al., 2023; Safarzadeh et al., 2024) (i.e., placenta and fetal membrane) and investigate the effects of therapeutics prior to human trials (Saha et al., 2021; Shojaei et al., 2021); 4) creating models to evaluate fetal risk assessment of after maternal toxicant exposures (Kim et al., 2022; Richardson et al., 2019c); 5) modeling inflammatory responses to bacterial infection [i.e., chorioamnionitis, ascending urea plasma parvum infection (Tantengco et al., 2022b; Zhu et al., 2018; Richardson et al., 2020b; Bento et al., 2023)] and adverse pregnancy outcomes (Tantengco et al., 2021; Lee et al., 2016; Mosavati et al., 2020; Zhu et al., 2018).

Progress has been made in recent decades regarding women’s health and basic and translational research in obstetrics. However, much work utilizing these new approach methods is still needed as dictated by the alarmingly high rate of pregnancy complications. Pregnancy is a complex system due to the interaction of two unique physiologies, the mother and the fetus, and much is still unknown regarding the inner workings of this multifaceted reproductive process. The challenges posed by the many limitations of animal models, current *in-vivo* and *ex-vivo* study designs, and ethical concerns associated with the direct investigation of pregnancy in human subjects hinder the depth of understanding one can obtain through research. OOC devices can accelerate research in this area and advance the understanding of pregnancy health and adverse pregnancy events by more accurately emulating the complexity of human reproduction in experimental settings. In this review article, we discuss the current methodologies and limitations of reproductive and obstetrics-based OOC platforms and highlight the advances in high throughput data acquisition techniques that will enhance the biological and translational potential of data collected from microfluidic experiments.

## Current OOC methodologies and limitations

### Device fabrication and fluid dynamics

Though OOC platforms more accurately represent human anatomy and biological function than current *in-vitro* systems, they do have limitations hindering their utility in different scientific fields (i.e., bench research, toxicology, and pharmacology). These restrictions include fabrication materials, modeling vascular flow, and the lack of biologically relevant multi-organ systems. The sections below will elaborate on this list of limitations and the challenges in overcoming them with current technological approaches.

## Materials

**FIGURE 1:**
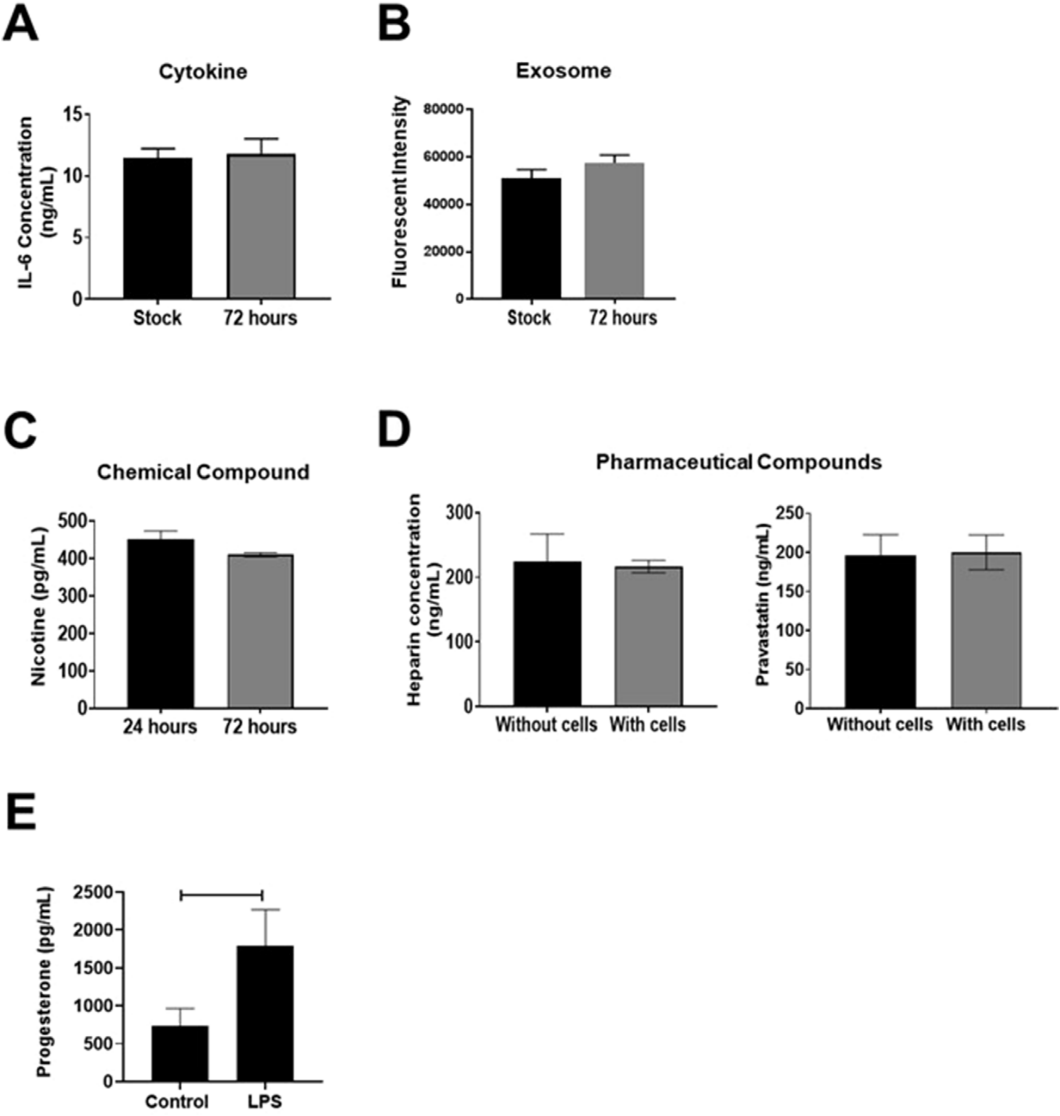
PDMS adsorption of compounds. **(A, B)** ELISA measured pro-inflammatory cytokine Interleukin (IL)-6 concentrations or CSFE stained exosomes within an OOC device, showing no difference over a 72-h period, indicating no or limited adsorption to the PDMS device or reservoir system (n = 3). Values are expressed as mean ± SEM. This figure is adapted from Richardson et al. (2020b) and Radnaa et al. (2021). **(C, D)** Targeted mass spectrometry analysis measured nicotine, heparin, or pravastatin concentration within an OOC device. Nicotine levels did not change over 72-h, and heparin, nor pravastatin concentrations differed after 8-h in devices with or without placental cells (n = 3). Values are expressed as mean ± SEM. Figures are adapted from Richardson et al. (2022), Richardson LS. et al. (2023). **(E)** ELISA-based evaluation of progesterone adsorption within OOC devices. Within a choriodecidua OOC, maternal decidua LPS treatment induced significant progesterone production within the neighboring chorion cells (p = 0.04). Values are expressed as mean ± SEM. The figure is adapted from Richardson et al. (2023a).

Polydimethylsiloxane (PDMS) is one of the most used materials in microfluidics as it allows easy prototyping through simple soft lithography techniques (Gokaltun et al., 2017). By casting PDMS prepolymer mixed with the curing agent onto a master mold, replicas with designed structures can be fabricated after incubation in an oven. The PDMS-enclosed microenvironment is supportive of cell growth due to its biocompatibility and gas permeability (Shakeri et al., 2021). Most importantly, PDMS is transparent to visible light (Dizon et al., 2019) and its low autofluorescence (Gokaltun et al., 2017) allows bright-field and fluorescent microscopy to monitor cell morphology and various marker expressions. The major disadvantage of PDMS is the adsorption of lipophilic proteins and small molecules (e.g., estrogen) because of the hydrophobic surface (Dizon et al., 2019; Ren et al., 2010). Recent studies related to reproductive OOCs have shown that PDMS devices do not adsorb hydrophilic compounds such as cytokines (Richardson et al., 2020b) (i.e., Interluken-6), biological signalers (Radnaa et al., 2021) (i.e., exosomes), endocrine factors (progesterone), chemical compounds (Richardson LS. et al., 2023) (i.e., nicotine), nor pharmacological compounds (Richardson et al., 2022) (i.e., Pravastatin, Rosuvastatin, Aspirin, Heparin), and environmental toxicants [i.e., cadmium (10µM ∼ 20% adsorption) (Kim et al., 2022)] (Figure 1). It is important to identify OOC material’s constraints, and pilot studies should be set up to determine the adsorption properties of analytes under investigation. For example, though PDMS is suspected of adsorbing endocrine factors, our models have shown that cells grown on-chip still produce functionally active naïve progesterone and progesterone synthesis can be inhibited by pathologic agents (i.e., lipopolysaccharide) (Richardson et al., 2023c) (Figure 1E). We were also able to test the functional effect of progesterone on cells grown on chips by disturbing its receptors (Lintao et al., 2024). These results highlight the detectability of high concentrations of biologically relevant progesterone even in the wake of slight adsorption by PDMS.

Many approaches have been suggested and studied to increase the hydrophilicity of PDMS surface to reduce PDMS-to-compound binding. First, oxygen plasma treatment can increase the surface energy on PDMS quickly without affecting the transparency for imaging, but the plasma effect declines rapidly and is lost within hours (Miranda et al., 2021). To achieve long-term treatment, additional coating with a zwitterionic polymethacrylate copolymer has been proposed, in which the hydrophilic surfaces could last for at least 200 h (Dizon et al., 2019; Miranda et al., 2021). Investigation on using 5% Pluronic F127 coating on PDMS devices to block non-selective adsorption of hydrophobic small molecules has been done and shows that the Pluronic F127 layer could effectively separate the interaction between PDMS surface and culture media and reduce the adsorption of Nile Red without affecting cell viability (Hawkins et al., 2022). Instead of treating the surfaces of PDMS after curing, Gökaltun et al. suggested mixing liquid PDMS with a smart copolymer additive containing PDMS as the base elastomer and 60%–70% hydrophilic polyethylene glycol (PEG) before curing (Gokaltun et al., 2017). This copolymer additive self-organizes on the surface when exposed to water and has the PEG segment in contact with the aqueous environment to prevent adsorptions of small molecules without additional steps, while the hydrophilic property could last for 20 months (Gokaltun et al., 2019). Thermoplastics such as polycarbonate, polymethyl methacrylate, and polyvinyl chloride are favorable in manufacturing for scale-up production (Shakeri et al., 2021; Miranda et al., 2021) using injection molding, carbon dioxide laser cutting, and the adsorption of small molecules could be avoided (Gokaltun et al., 2017; Busek et al., 2021). However, it requires more complicated equipment and expensive molds for developing new devices (Sonmez et al., 2020), which might not be a suitable approach for prototyping. Additionally, many of these methods restrict the number of cell culture chambers that can be fabricated within an OOC platform, limiting their ability to recreate anatomically correct organ structures. Further research is needed to validate and assess a variety of fabrication materials and their utility in housing multiple cellular and collagen layers to create physiological OOC devices.

## Modeling fluid flow

In OOC platforms, media is perfused into a cell culture chamber by either a syringe pump and peristaltic pump perfusion (Novak et al., 2020a; Wang et al., 2018; Hsieh et al., 2019; Novak et al., 2020b) or by gravity feed media reservoirs (Richardson et al., 2019a; Richardson et al., 2020b; Richardson LS. et al., 2023; Phan et al., 2017; Wang and Shuler, 2018; Esch et al., 2016). Media perfusion is most common as it serves to mimic the circulatory system that transports sufficient nutrients for long-term cell culture and recapitulates a better physiologically relevant micro-environment compared to a static culture system for organs that contain physiological blood flow (Gijzen et al., 2021; de Graaf et al., 2022). Unlike conventional blood flow *in-vivo*, modeling *in-utero* fluid dynamics (i.e., placental blood flow or amniotic fluid movement) is challenging. Maternal to fetal blood flow at the placental interface is specifically challenging to model within pregnancy-related OOCs due to the numerous lobules of the placenta and maternal spiral arteries that are inter-branched, allowing for non-unform slow blood flow. As it is difficult to calculate the exact amount of fluid flow and shear stress experienced by the placental cells *in-utero*, researchers have utilized arbitrary fluid flow values (i.e., 50–100 μl/h) (Pemathilaka et al., 2019; Blundell et al., 2018; Blundell et al., 2016) to create low levels of fluid perfusion. Though fluid flow across placental trophoblast cells has been shown to upregulated microvilli formation (Miura et al., 2015; Blundell et al., 2016), it is unknown if any negative or non-physiological effects are present due to these conditions. In addition, the amniotic fluid that surrounds the fetus slowly moves back and forth due to maternal and fetal movement, resulting in low levels of shear stress on the amnion epithelial cell (Morandi et al., 2023; Richardson et al., 2020c). However, studying this motion has been limited due to the lack of proper *in-vitro* platforms. Kim et al. recently studied the effect of amniotic fluid on amnion membrane disruption using an existing OOC by slow shaking (Richardson et al., 2019a; Kim et al., 2024). Programmable syringe pumps with the desired flow rate were connected to the device to control the flow direction and time interval. However, the experimental setup has not fully mimicked the *in-vivo* situation as it only created back-and-forth fluidic movement, while the physiological situation is much more complicated, including vibration and random influx and outflux of fluids. Integrating electrical or mechanical engineering modules to improve the system should be considered to recreate a higher degree of physiological properties and functions of targeted tissues or organs. Real-world validation techniques, such as using patient-derived tissues or microvascular modeling, could be helpful to try and tease out proper flow rates that should be incorporated into these key interfaces.

### Cellular components

Traditionally, to establish biologically relevant physiologic or pathologic states on-chip, cellular components are assessed by a variety of standard molecular assays to determine functional relevance to the human organ of interest. Cellular components are either kept on-chip and analyzed for morphologic, cytotoxic, or migratory changes (bright-field microscopy or live imaging), protein expression (Immunocytochemistry [ICC]), or barrier function (transendothelial electrical resistance [TEER]). Collection of cells, or cellular components, from devices through trypsinization, cell lysis, or TRIzol allows protein analysis by Western blot, gene expression by RT-PCR, or cell fate and marker analysis using flow cytometry.

## Cell availability

The ability of human primary, immortalized cells or iPSCs grown in OOC platforms to biologically recreate human physiology is one of the most significant advancements of this methodology in the field of science. However, when it comes to recreating reproductive or pregnancy-related organs, there are many hurdles in acquiring primary tissue or cell lines for use within said platforms, especially since iPSC lines are not available for many organs of interest nor iPSCs will not undergo the natural aging of fetal organs as expected *in-utero*. Few labs obtain local access to reproductive or pregnancy-related tissue to derive primary cells, and fewer have the resources to establish validated cell lines from said organs. Thus, restricting the use of physiologically accurate cells to be cultured within OOCs. Furthermore, commercially available cell lines do not always mimic the *in-vivo* cell type they are derived from, or they are collected from carcinomas or diseased tissue that could affect their functional responses on-chip. Often these cellular restrictions force scientists to use improper cell lines (e.g., BeWO, JEG, WISH cells) or to exclude cell types altogether due to the current state of the field’s resources. This can often be seen with commercial platforms that only allow for two cell culture chambers and are trying to recreate complex multi-layered tissue. This cellular challenge is most detrimental, as it can lead to biologically inaccurate results and cast doubt on OOCs within different fields.

## Cellular assays and chip-related challenges

### Microscopy

Bright-field and fluorescence microscopy are basic optical microscopy technique that uses transmitted light and filter cubes to produce images of a sample, commonly used to visualize the structure of cells and tissues and observe morphological changes (Tantengco et al., 2021; Gnecco et al., 2019; Gnecco et al., 2017; Kreuder et al., 2020; Rogers et al., 2018; Richardson L. et al., 2020; Richardson et al., 2020b; Kim et al., 2022). It is simple, inexpensive, and can be combined with other techniques. Bright-field microscopy can be used in OOC studies to visualize live cell and their structure, assess cell health and function, and monitor morphological changes in response to stimuli. This technique can provide important information to optimize conditions and improve understanding of organ responses in a controlled, reproducible manner. However, microscopy-related results only provide a “snapshot” of one timepoint and one region within a device leading to unwanted bias when assessing cell morphology endpoints. To overcome this limitation, many groups have turned to live cell imaging to evaluate cell responses and movement in real-time. This technique is a valuable method for visualizing cells and their adaptations to stimuli in real-time within a microscale environment such as OOC devices (Yu et al., 2022; Nagashima et al., 2018; Park et al., 2022; Boos et al., 2021; Richardson et al., 2020b). Boos et al. used live imaging to monitor placental proliferation and growth in the development of their microfluidic device that models the embryo-maternal interface (Boos et al., 2021). This provides valuable information on cellular responses to various stimuli and enables the development of more accurate organ models.

### Immunocytochemistry (ICC)

ICC is a laboratory technique to visualize and analyze the distribution and localization of specific proteins or other cellular components in fixed cells or tissues. It uses antibodies that bind specifically to target antigens and visualizes the binding with a detectable label, such as a fluorescent dye, to obtain information about the presence and location of the target within the cell or tissue. ICC can be used in OOC studies to analyze the distribution and localization of specific cellular components (Richardson et al., 2019a) within the cells and tissues on the chip (Xiao et al., 2017; Ahn et al., 2021; Park et al., 2022; Mosavati et al., 2020; Pemathilaka et al., 2019; Yin et al., 2019; Blundell et al., 2018; Abostait et al., 2022a; Mandt et al., 2018; Ghorbanpour et al., 2023; Pemathilaka et al., 2022; Saha et al., 2020), providing insight into cellular and molecular functions (Baert et al., 2020; Tantengco et al., 2022a; Tantengco et al., 2021; Tantengco et al., 2022b; Saha et al., 2021; Park et al., 2020; Gnecco et al., 2019; Gnecco et al., 2017; Kreuder et al., 2020; Boos et al., 2021; Blundell et al., 2016; Rogers et al., 2018; Richardson et al., 2020b; Kim et al., 2022) and advancing the development of these devices for various applications such as disease modeling and drug screening. Mahajan et al. used immunostaining on their vagina-on-a-chip to visualize the presence of cytoskeletal proteins (i.e., Cytokeratin [CK]14, CK15, CK5, CK13, F-actin), junction markers (i.e., Involucrin, Zonula occludin-1, E-cadherin), and desmosomes (i.e., Desmoglein [DSG]-3, DSG-1) in the squamous stratified vaginal epithelium (Mahajan et al., 2022). ICC is limited, however, in the number of fluorophores (i.e., markers) that can be evaluated at one time. Traditional fluorescent microscopy allows four excitation/emission cubes to simultaneously evaluate four tagged antibodies. This still restricts the throughput of analysis conducted within cell culture chambers.

### Transendothelial electrical resistance (TEER)

The on-chip measurement of cell barrier function using TEER measurement is very commonly used approach. Blundell et al. confirmed the integrity of OOC in their placental drug transport studies using TEER, which assessed the structural integrity of the placental barrier (i.e., trophoblast-endothelial layers) and its ability to mimic drug transport across the maternal-fetal interface in the human placenta (Blundell et al., 2018). This method provides a quantitative determination of the barrier function of cell cultures for OOC experiments to assess system functionality, drug toxicity, and efficacy (Gnecco et al., 2017; Yin et al., 2019; Blundell et al., 2018; Boos et al., 2021). However, current TEER machines provide “chopstick” style electrodes that are only compatible with transwell-orientated OOC platforms. This greatly restricts the usability of this methodology across OOC systems. Advanced engineering of TEER electrodes etched on the glass surfaces of the OOC can record TEER in real-time, overcoming the challenges of the current TEER platforms.

### Real-time PCR (RT qPCR)

To analyze target RNA in a cell sample, RT qPCR is commonly used by combining the principles of PCR with real-time fluorescence detection. It is a rapid, sensitive, and specific tool used in molecular biology and diagnostics. RT qPCR can be used to assess gene expression in cells grown on OOC in microscales cultures. Data can be used validate functional and molecular similarities to *in-vivo* situations, providing insights into gene regulation and cellular responses (Baert et al., 2020; Mahajan et al., 2022; Tantengco et al., 2022b; Nagashima et al., 2018; Saha et al., 2021; Park et al., 2020; Ahn et al., 2021; Aziz et al., 2020; Yin et al., 2019; Saha et al., 2020). It can also be used for quality control. Yin et al. used qPCR to evaluate the expression of Iinterluken-6 (an inflammatory cytokine) secretion from the system after nanoparticle exposure to investigate the *in-vitro* experimental placental response in a pathological condition (Yin et al., 2019). Unfortunately, due to the low number of cells seeded within OOC chambers, collection of high abundance pure RNA is very challenging and limits the throughput of transcript analysis.

### Protein analysis via Western blot or flow cytometry

Western blotting, which is a semi-quantitative laboratory technique for analyzing specific protein markers in a sample by separating the proteins, can be used in OOC studies to analyze the protein expression profiles of cells collected from microfluidic devices. This provides insight into the health and function of the cells and the overall system (Tantengco et al., 2022a; Saha et al., 2021; Park et al., 2020). Park et al. used Western blotting to analyze the enhanced mRNA and protein levels of plasminogen activator inhibitor-2, a reliable reproductive toxicity marker, in endometrial and ovarian follicular cells cultured in their dual reproductive OOC device following toxic exposure (Park et al., 2020). Conversely, semiquantitative protein marker analysis can be conducted through flow cytometry, that analyzes the physical and chemical characteristics of individual cells in a fluid stream by using lasers and fluorescence to detect and profile the cells based on their size, shape, contents and other parameters (Saha et al., 2020). It is commonly used in OOC experiments to allow real-time analysis within the system to quantify cells in the system, assess cell viability and phenotype, and measure the secretion of specific cytokines or other biomarkers that may indicate proper organ function (Park et al., 2020; Park et al., 2022; Boos et al., 2021; Kim et al., 2022). This technique was used by Kim et al. to analyze the cell cycle of maternal decidua cells and determine the effect of cadmium exposure on cell proliferation inside their feto-maternal interface organ-on-chip (FMi-OOC) (Kim et al., 2022). However, a general limitation of microfluidic platforms is the use of minimal amounts of cells (i.e., 50,000–100,000 per chamber) to establish the organ system. While this can increase the throughput of devices that can be run in a single experiment and minimize reagent costs, it also restricts the type of endpoint assay that can be conducted without pooling time points or devices (e.g., flow cytometry and traditional Western blotting).

## Analyzing supernatants

### Enzyme-linked immunoassay (ELISA)

ELISA is a type of assay that uses an enzyme-labeled antibody to detect and quantify the presence of a specific protein in a sample (Xiao et al., 2017). It is commonly used for detecting and quantifying hormones, cytokines (Mahajan et al., 2022; Richardson et al., 2019a; Saha et al., 2020), and antigens (Baert et al., 2020; Xiao et al., 2017; Tantengco et al., 2022a; Mahajan et al., 2022; Tantengco et al., 2022b; Aziz et al., 2017; Gnecco et al., 2019; Gnecco et al., 2017; Park et al., 2022; Aziz et al., 2020; Abostait et al., 2022a; Blundell et al., 2016; Rogers et al., 2018; Richardson et al., 2020b; Kim et al., 2022). Xiao et al. used ELISA to detect ovarian hormone production, Interluken-8, vascular endothelial growth factor, and albumin between microfluidic and static cultures (Xiao et al., 2017). The limitation of this methodology lies in the required supernatant volume needed for analysis (i.e., ∼100 μl per analyte), which utilizes a majority of one cell chamber’s media volume. To overcome this, researchers have turned to Luminex multiplexing, which is a multiplexed immunoassay system used to simultaneously detect the presence of many specific proteins or other analytes by capturing specific targets onto spherical beads in suspension (Tantengco et al., 2022a; Tantengco et al., 2021; Tantengco et al., 2022b; Saha et al., 2021; Richardson et al., 2020b; Kim et al., 2022). Luminex multiplexing technology can be used in OOC studies to simultaneously measure multiple biological markers (i.e., up to 20–40 analytes depending on their compatibility within kits) in small fluid volumes (i.e., ∼25 µL) collected from chips (Baert et al., 2020; Xiao et al., 2017; Tantengco et al., 2022a; Tantengco et al., 2021; Tantengco et al., 2022b; Aziz et al., 2017; Saha et al., 2021; Gnecco et al., 2019; Gnecco et al., 2017; Park et al., 2022; Abostait et al., 2022a; Rogers et al., 2018; Richardson et al., 2020b; Kim et al., 2022). This provides valuable information about the health and function of the cells and tissues, which can be used to optimize the performance of these devices.

### Mass spectrometry

Targeted mass spectrometry is an analytical tool that measures the mass-to-charge ratio of charged particles, allowing for an in-depth identification of a specific subset of compounds within a complex sample. It allows for highly sensitive and selective quantification of targeted analytes, providing more specific information than untargeted mass spectrometry approaches. Targeted mass spectrometry can be useful in OOC studies as it can analyze biological markers in small fluid volumes collected from the chip, allowing researchers to understand cellular responses and biomolecular interactions (De Bem et al., 2021; Kim et al., 2022). For example, Park et al. used mass spectrometry to quantify proteins from conditioned media on an implantation-on-a-chip to explore the established functions of the proteins found in relation to implantation and placentation and to discover new proteins that may have an unknown role in these processes (Park et al., 2022). Pemathilaka et al. (2022) used liquid chromatography/mass spectrometry (LC-MS) to measure Naltrexone concentrations, a drug commonly used for opioid treatment to determine if it crosses the placenta.

## New technologies primed to increase data generated from OOCs

**FIGURE 2:**
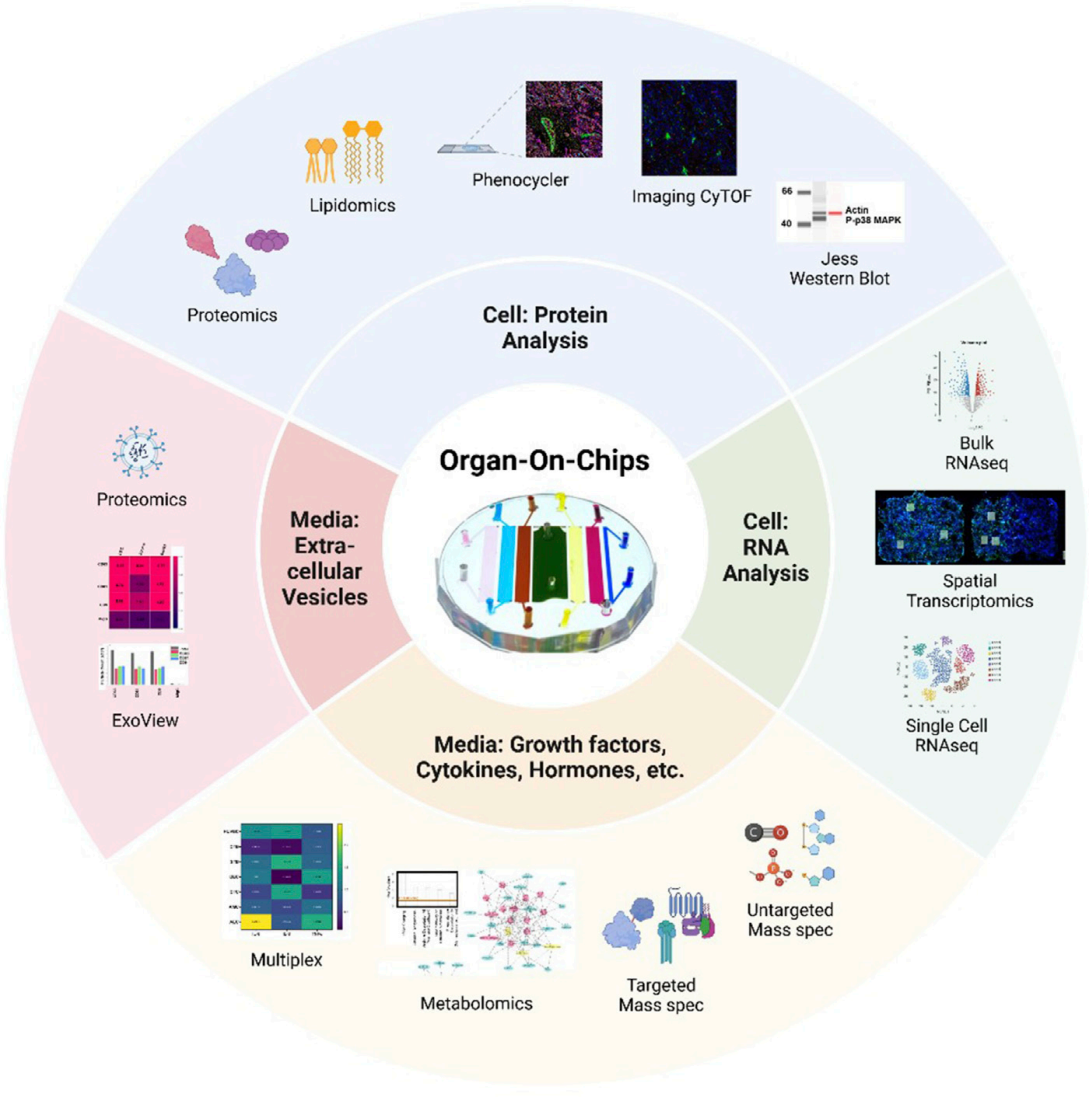
Summary of advanced methodologies to study cells and media from organ-on-chips A variety of protein analysis strategies can be used to assess cell phenotypes on-chip, including proteomics, lipidomic, PhenoCycler or imaging CyTOF multiplexing of antibody staining, or Jess Western blotting. RNA collected from OOCs can be analyzed by bulk or single-cell RNAseq, while spatial transcriptomics allows for regions of interest (white boxes) to be selected from cell on-chip. Media collected from individual chambers can be analyzed for a variety of secreted factors by multiplexing for specific targets or by conducting mass spectrometry for metabolites, targeted, or untargeted molecules. Extracellular vesicles, like exosomes, can be evaluated from small volumes of OOC media by methods such as ExoView or proteomics. The device in the center is the multi-organ fetal membrane and placenta feto-maternal interface OOC (Kammala et al., 2023; Safarzadeh et al., 2024).

The limitations described above have started to be addressed with novel molecular and biological methodologies that specialize in using low numbers of cells and supernatants to generate libraries of data. However, these novel techniques are only utilized by a minority of OOC platforms, and therefore, to provide examples of each technique’s utility, the below section will describe results generated from pregnancy-related OOCs. These sections will be divided by RNA and protein analysis of cells and supernatants (Figure 2).

### Strategies for generating high throughput RNA data from OOC-derived cells

**FIGURE 3:**
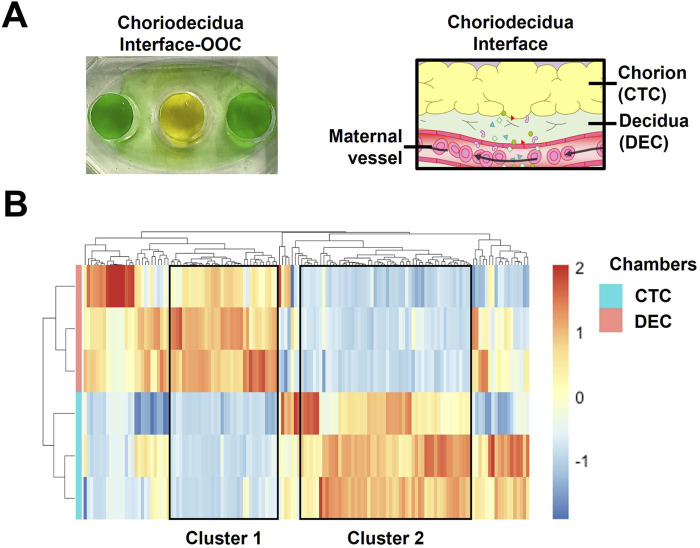
Utilizing targeted RNAseq to characterize the chorion and decidua cells on-chip **(A)** Color dye image of a two-chamber chorio-decidua interface OOC and the corresponding anatomical structure. **(B)** Heatmaps of transcripts involved in inflammation and immunity with >1,000 reads identified in chorion trophoblasts (n = 3) and decidual stromal cells (n = 3) derived from the chorio-decidual interface-on-chip. Transcript intensities were shown as z-scores and were displayed as colors ranging from red (highest) to blue (lowest). The rows and columns are clustered using correlation distance and average linkage. The figure is adapted from Lintao et al. (2024).

As mentioned above, current OOC users face multiple challenges when trying to generate transcriptome-based cellular data. Specifically, difficulties in isolating high quantity, pure RNA and problems collecting cells out of cell culture chambers are quite common. Two high throughput technologies, RNAseq and spatial transcriptomics (Figure 2), can help to alleviate these challenges. RNAseq is a technique that uses next-generation sequencing to produce transcriptome data (e.g., pooled RNA snapshot of the cells’ status) from cellular samples (Wang et al., 2009). In general, RNA isolated from chips are converted into cDNA library fragments with adaptors, amplified, and sequenced in a high throughput manner to generate reads through multiple platforms (i.e., Illumina IG, Applied Biosystems SOLiD). This allows for high-throughput and complex analysis even with low RNA yield arising from generally low cell numbers in OOC platforms. This technology has recently been utilized with chorion and decidua-derived RNA from the chorio-decidua immune interface OOC (Lintao et al., 2024). This library of transcriptome data generated a heatmap of inflammation (e.g., Toll-like receptors, interferons, chemokines) and immunity genes (e.g., clusters of differentiation, human leukocyte antigens) that were differentially expressed in chorion trophoblasts and decidual stromal cells (Figure 3). It provides a large RNA library that can now be investigated for future biological implications highlighting its application within OOC platforms. Sometimes, cells are extremely difficult to remove from cell chambers. Planar OOC devices can be adapted to use spatial transcriptomics methodologies that enable researchers to spatially localize and quantify gene expression on-chip. This technology was recently used to select targeted regions of *in-vivo* tissue or *in-vitro* cell culture, compare the transcriptomic profile of human decidua to decidual cells grown within an OOC device surrounded by other pregnancy-related cellular layers. This type of analysis allows researchers to specifically select regions of interest within OOC platforms or tissues and conduct full transcriptome analysis without removal of the cells from their biological context.

### Generation of OOC cell-derived high throughput protein data

**FIGURE 4:**
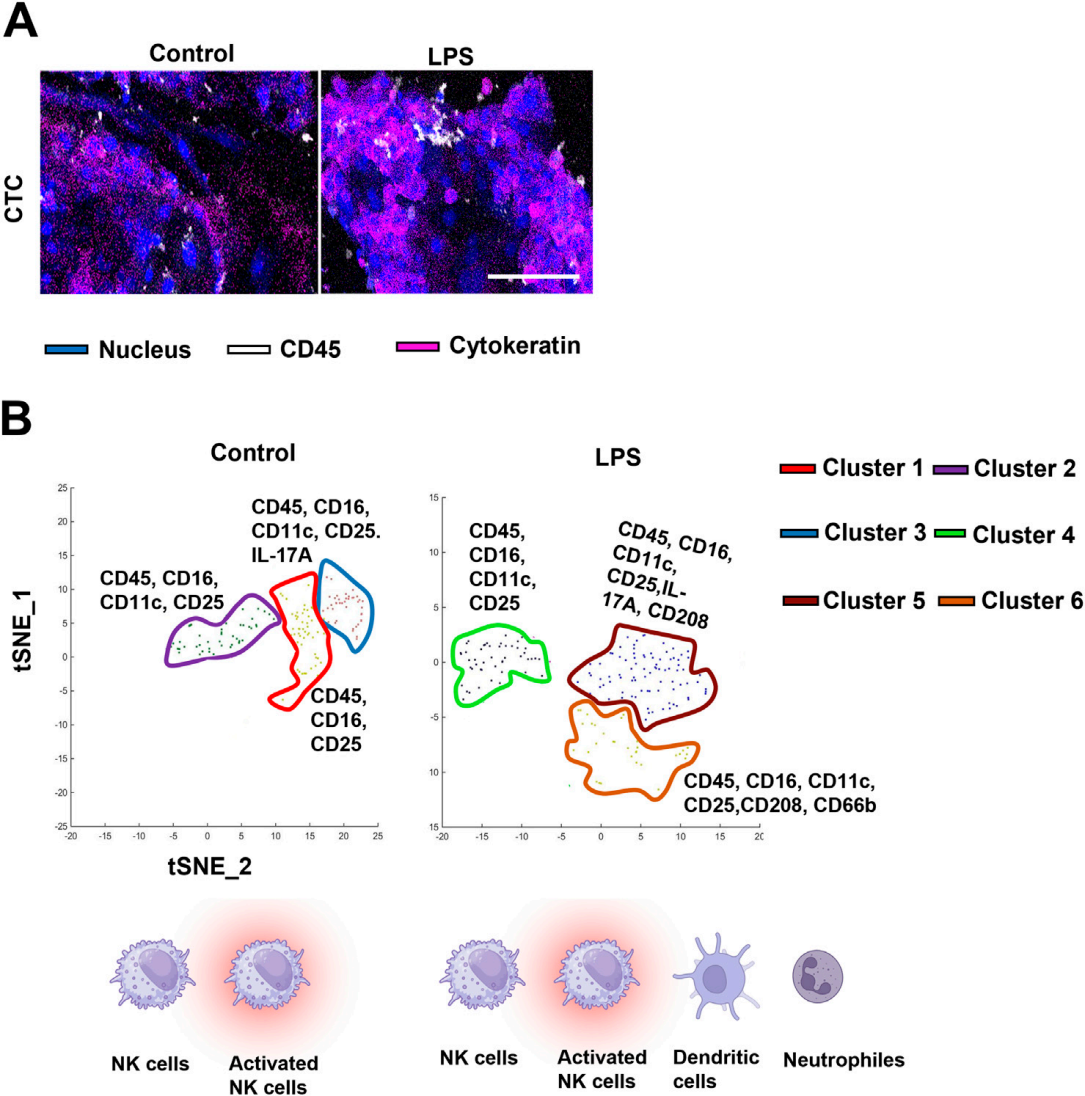
Adapting OOC devices for imaging CyTOF analysis of immune cell activation at the choriodecidua interface **(A)** CyTOF images showing migratory leukocytes identified by their CD45 staining (white) next to chorion cells (blue nuclear stain and pink cytokeratin stain) in the center chorion chamber. Scale bars, 100 µm. **(B)** Phenographs overlaid on t-SNE plots showing the specific immune cell clusters identified in each group. Different colors represent different histoCAT-identified cell clusters which were dominant in differentially expressed immune cell markers. Background clusters that did not meet the inclusion criteria were removed from these graphs. The figure is adapted from Richardson et al. (2023a).

In parallel with RNA analysis, protein-expression data is often necessary to determine disease states or decipher the biological implications of differential expression of transcripts. Protein data derived from cells or media from OOC experiments face limitations as described above. However, several novel technologies can overcome these challenges to derive the protein signature. These technologies include both on-chip (i.e., imaging CyTOF or PhenoCycler) and off-chip (i.e., proteomics and Jess Simple Western blotting) platforms (Figure 2). Multiplex protein analysis of cells on-chip is only accomplished by imaging CyTOF which is an application of mass cytometry used to quantify metal-labeled targets (i.e., up to 30) on the surface and interior of single cells. Cells are immunostained with pre-conjugated metal antibodies that are then laser ablated, collecting protein data for downstream analysis. Recent experiments utilizing the chorio-decidua immune interface OOC assess the status of migratory primary immune cells into the chorion chamber using this approach (Richardson et al., 2023c). Figure 4 shows a subset of these data highlighting imaging CyTOFs ability to identify and characterize unique immune cell sub-types on-chip (i.e., NK cells, dendritic cells, and neutrophils) after control or lipopolysaccharide (LPS) treatment (Figure 4B). These results emphasize the unique ability of this technology to document multiplexing of protein-level data without removing cells from their culture chambers.

**FIGURE 5:**
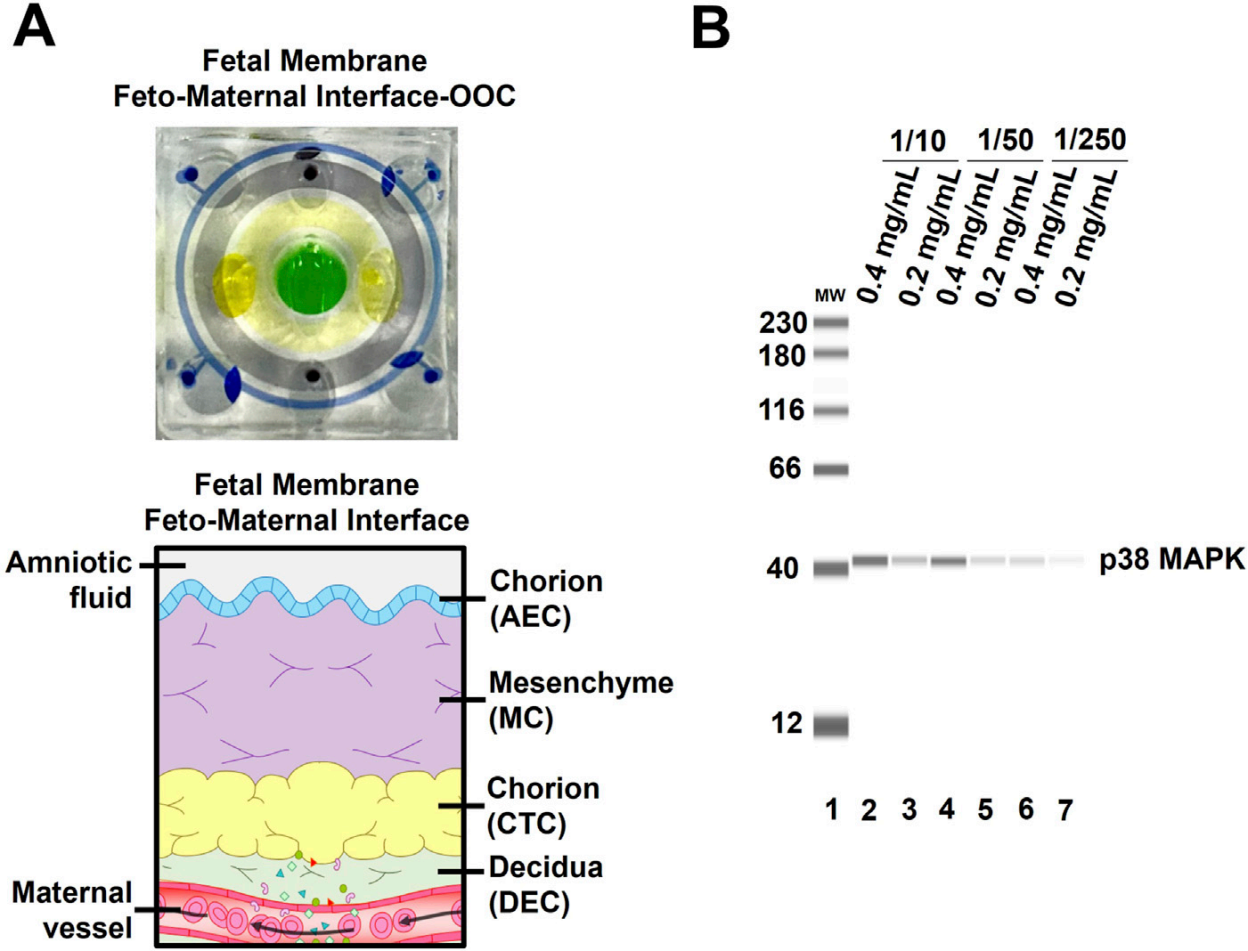
Quantifying protein expression from individual devices and various cells within OOC platforms **(A)** Color dye image of a four-chamber fetal membrane feto-maternal interface OOC and the corresponding anatomical structure. **(B)** Protein analysis using Jess Simple Western blotting platform. Optimization of protein quantity per lain (i.e., 0.2–0.4 mg/mL) and p38 MAPK antibody titration. The figure is adapted from Bento et al. (2023).

If collection of cellular components is possible from chip experiments, two sensitive methodologies can be implemented to screen for overall protein profiles using mass spectrometry and eukaryotic libraries (i.e., proteomics), followed by Jess Simple Western blotting to confirm protein expression in a multiplex scale using microliters of sample. Proteomic analysis is possible from single-cell chambers of most OOC platforms that contain around 100,000–200,000 cells (i.e., ∼9–15 μg of total protein), though recovery rates differ across devices. Isolation methods vary greatly depending on local expertise or sup-types of downstream mass spectrometry analysis; however, different styles of isolation (i.e., RIPA lysis then buffer transfer into TEAB or isolation with TEAB) greatly affect differential protein expression and should be standardized across experiments. To further confirm these protein results, automated Jess Simple Western blotting can evaluate protein expression using minimal sample volume (2–5 μl; i.e., typically 0.2–0.4 mg/mL of protein) (Bento et al., 2023) (Figure 5). This analysis can be extended to incorporate antibody multiplexing with chemiluminescence and fluorescent-based antibodies. Thus, a novel method for protein-level Western blot analysis within single-cell chambers in a single OOC device is established.

### High throughput analysis of OOC-derived cell supernatants

Depending on the OOC platform, supernatant collection and volumes (i.e., small volume size or over-diluted) can limit the number of endpoint assays accomplished at a single time point within one device. While multiplexing of cytokine and hormone analysis is quite common to overcome these limitations (i.e., requiring 25 μl of sample to assess 5–40 analyte, instead of 100ul per analyte), other methodologies are available to generate additional high throughput data from small volumes. One such approach is utilizing mass spectrometry to assess proteins, secreted metabolites, or lipid profiles in an untargeted manner and then comparing these results to documented proteomics, metabolomic, or lipidomic libraries (Figure 2). By utilizing “multi-omics” approaches, a biological profile can be generated from each cell layer within OOC devices, contributing to the establishment and validation of these systems. Ongoing studies suggest this type of assessment can be conducted on a single-cell chamber to investigate a particular cell layer’s function or metabolism of a drug/toxicant or utilized to investigate the overall organ’s response. Though these results document normal metabolic pathways and processes found within these cellular layers, targeted metabolic approaches can be used to document drug or toxicant propagation and metabolism to generate translationally impactful data from low sample volumes.

Over the past 10 years, exosome signaling at the organ and cellular level has been documented to play critical roles in endocrine or paracrine signaling and the development of disease states. Exosome analysis is typically restricted to media samples greater than 500ul in volume; within those samples, the quality and quantity of exosomes are minimal. ExoView™ R100 platform is an immuno-affinity-based technology that allows specific exosome populations (i.e., CD63, CD81, and CD9) to bind in a multiplexed manner to a functional ExoView™ chip (Figure 2). The instrument provides multi-level and comprehensive exosome measurements for particle size analysis, and phenotyping (i.e., CD markers). Ongoing studies document that 100 μl of samples collected straight from OOC cell chambers (i.e., “unisolated” and cultured in fetal bovine serum-free media) can be analyzed using ExoView™ chip to determine exosome quantity and characterize CD marker expression. Immunostaining for proteins of interest within the lumen or membrane of exosomes is also possible with this technology (Kammala et al., 2022). Still, there are challenges with exosome research on OOCs. Downstream characterization of exosome cargo using proteomics and other OMICS or additional functional studies can be challenging as the chips may not yield a very high quantity of exosomes.

## Utility and future of OOC technology

**FIGURE 6:**
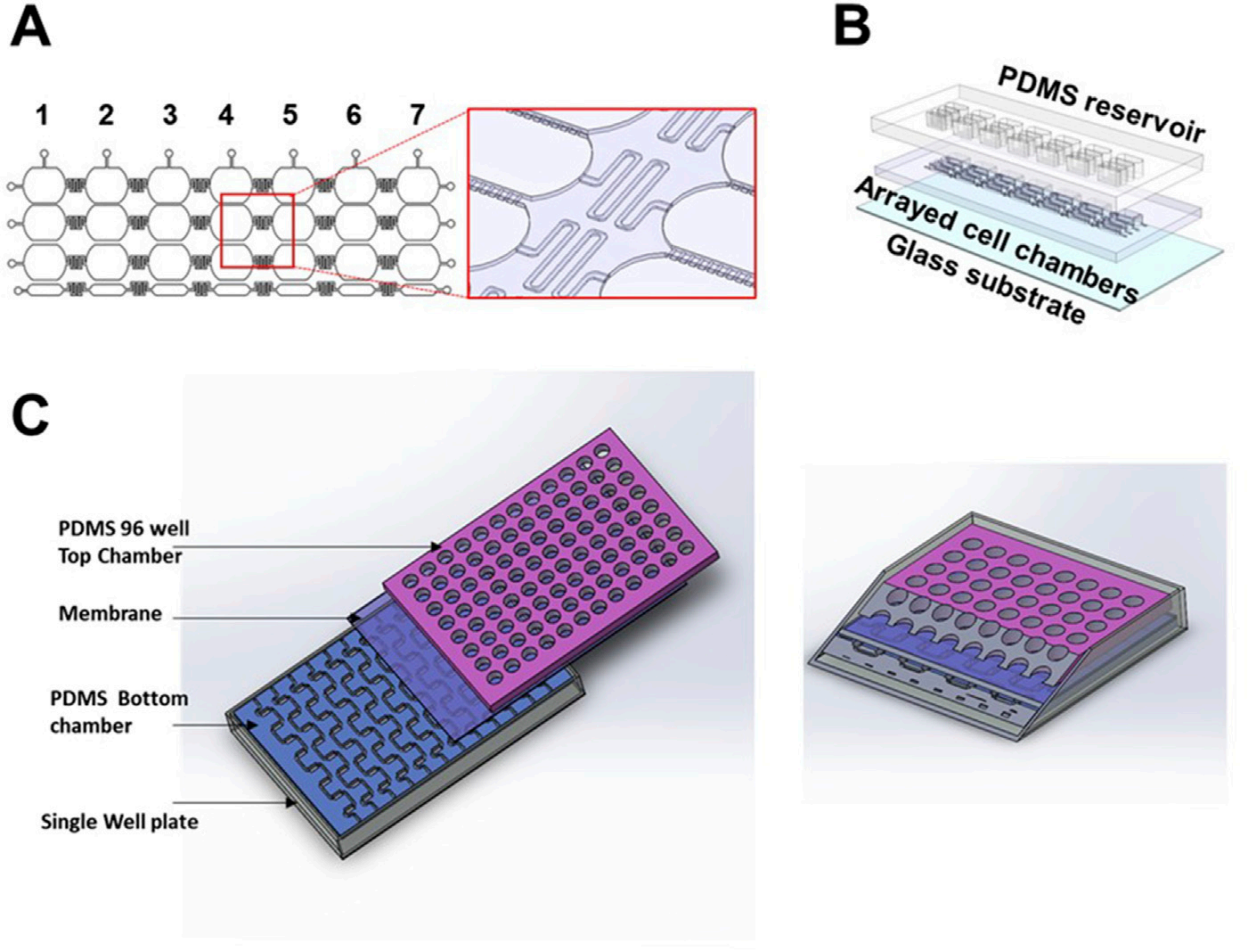
Development of array OOC platforms to model the fetal membrane feto-maternal interface **(A)** 2D and 3D schematics of an arrayed OOC device used to model the feto-maternal interface. The cropped region highlights the delay channels connecting each of the seven devices and microchannels connecting each cellular chamber within said device. **(B)** 3D drawing of the individual components of this array platform. **(C)** Highlights the concept of a vascularized feto-maternal interface model with cross-section.

OOC technology offers improved cell culture capability to create physiologically more relevant human tissue environments, leading to better predictive *in-vitro* models. However, creating *in-vitro* human physiology is often made at the expense of throughput, whether low throughput regarding the number of devices used in an experiment or low throughput regarding low data collection. Therefore, creating next-generation OOCs that allow for sufficient data collection requires novel methodologies (described above) merged with advanced microfluidic engineering architecture together with the corresponding biology. Scaling down OOC size (i.e., Mimetas placenta chip) (Rabussier et al., 2023b) or interconnecting arrays of devices, as shown in Figure 6, can be two alternative design strategies to increase the experimental throughput of OOC platforms. In arrayed models, multiple compartments are interconnected through microfluidic channels to operate multiple assays simultaneously, which enables semi-independent drug treatment (Figures 6A,B). A recent study has introduced a high throughput platform for drug development (Azizgolshani et al., 2021) that adapts this strategy. This platform supports multiple human tissue models, including liver, vascular, gastrointestinal, and kidney. In this system, 96 individual devices are integrated with programmable flow control systems for high throughput data collection. Electrical components, such as electrodes, are also integrated for electrochemical sensing within the design to produce higher readouts (Zhang et al., 2017; Bonk et al., 2015; Jin et al., 2020; Dornhof et al., 2022). Electrical sensors can also be embedded to quantify the barrier function through a real-time TEER (Bossink et al., 2021; Henry et al., 2017; Maoz et al., 2017; van der Helm et al., 2016b; Renous et al., 2021). Therefore, this system enables high throughput drug screening capability, increasing predictive accuracy.

Numerous biosensors have been developed and employed for a variety of sensing applications in microphysiological systems. The capability to obtain real-time measurements has enabled researchers to conduct experiments noninvasively and collect quantitative analytical data more efficiently. For example, impedance-based techniques have allowed for the study of single cells, providing insights into the cell cytoplasm through the membrane and enabling the investigation of cellular senescence (Xu et al., 2016).Additionally, oxygen sensors have been instrumental in detecting cellular oxygen deprivation and oxidative stress (Moya et al., 2018; Azimzadeh et al., 2021; Bossink et al., 2020). Various methods, such as aptamer-based tracking (Ferguson et al., 2013; Liu et al., 2015; Hao et al., 2018), electrochemical detection (Russell et al., 2019; Lu et al., 2021), and photonic biosensing (Cognetti et al., 2023), have been explored for real-time detection of inflammatory markers. These biosensors can also be integrated into the reproductive field to monitor the feto-maternal interface and predict preterm birth. Impedance-based sensors can assess the integrity of placental cells at the maternal-fetal interface. In contrast, oxygen sensors can monitor oxygen levels and identify potential hypoxia, a known risk factor for preterm birth. Moreover, various sensors capable of detecting inflammatory markers and hormonal levels (estrogen and progesterone) in real-time could be crucial for identifying intrauterine inflammation, which is a significant contributor to preterm labor. Yet, the integration of sensing technology to pregnancy-related applications has not been fully utilized as it requires complex fabrication processes with expensive core facility usage, significant effort on optimizing the sensors for desired sensitivity, thorough training of AI-driven systems to generate automated readouts, and consideration of biocompatibility. However, the field is making progress to integrate these systems into devices to enable continuous, noninvasive monitoring (Izadifar et al., 2024). Thereby making OOCs an effective tool for developing pathophysiological models and facilitating drug discovery.

Aside from biosensors, OOCs have started to reflect more complex physiology that is seen *in-vivo*. Among the advances in modeling the vasculature include the assembly of vascular cells into networks and their remodeling to form vascular beds underflow (Mandrycky et al., 2021). Optimizing the vasculature dynamics remains unresolved when creating a vascularized model of the placenta. This is particularly important in tissues and disease states where immune cells play a significant role in tissue homeostasis and pathogenesis (Van Os et al., 2023). In the context of pregnancy, given that labor is an inflammatory process, the inclusion of immune cells in pregnancy-related OOCs would better provide the immunological context of such a process, as seen in the chorio-decidual immune interface-on-chip experiments we have conducted (Richardson et al., 2023a). Current research is ongoing to develop a vertical, stacked, arrayed microfluidic device with vasculature on the bottom and a 96-well style array on the top for feto-maternal interface cell culture (Figure 6C). Overall, integrating vascular and immune cells into OOCs could potentially provide a mechanistic picture of physiologic and pathophysiologic processes seen *in-vivo*.

Utilizing high throughput methodologies and arrayed platforms is the future of the OOC field to meet the mandate stated by the FDA Modernization Act 2.0, which is expected to revolutionize the current state of pre-clinical drug testing. The FDA Modernization Act 2.0 allows for alternatives to animal testing (NAMs), such as OOC platforms or *in silico* simulation models, to generate drug-related data for regulatory purposes (Han, 2023; Haderspeck et al., 2019). This is especially relevant when it comes to developmental and reproductive toxicity (DART) studies and drug development for pregnant women, as pre-clinical studies are needed to provide safe, reliable, and reproducible data on drugs and to accelerate phase clinical trials for reproductive and pregnancy-related adverse outcomes. These experiments are essential as pregnant women are currently therapeutic orphans and are not included routinely in clinical trials (Illamola et al., 2018). Issues arising from DART testing are responsible for 10% of preclinical toxicology-related drug attrition, which in turn accounts for 20% of overall attrition during preclinical drug development (Guengerich and MacDonald, 2007). Currently, developmental and reproductive toxicity in pharmaceutical risk assessment is being done preclinically on two animal species, routinely rodents and rabbits (Stokes, 2015). Alternative models for reproductive and developmental toxicity, such as rodent whole embryo cultures, mouse embryonic stem cells, and zebrafish, have gained traction over the past decades (Brannen et al., 2016). These models are insufficient in generating data required by regulatory authorities for consideration of certain drugs, specifically therapeutics to be given during pregnancy (McCormack and Best, 2014; Adam et al., 2011; Sheffield et al., 2014). With OOC technology, researchers have been able to recreate reproductive tissues *in-vitro* that reflect *in-vivo* physiological processes. A microfluidic culture of murine ovarian follicles developed by Xiao et al., (2017) integrated various endocrine loops of the female reproductive tract to reflect a 28-day menstrual cycle hormone profile seen in human, which has potential implications on how potential drug candidates can disrupt hormonal profiles in females. Recently, a human testis-on-a-chip was able to demonstrate the reciprocal crosstalk between Sertoli and Leydig cells that are important in germ cell development and spermatogenesis (Park et al., 2024). Using this platform, researchers were able to identify SERPINB2 as a marker of male reproductive toxicity, which has implications for toxicity screening of potential drug candidates.

In addition to DART testing, OOC technology can be a solution to the lack of drug data in the pregnant cohort due to ethical concerns with including pregnant women in clinical trials (Young and Huh, 2021). Specifically, reproductive and pregnancy-related OOCs can be utilized at every stage of the drug discovery pipeline, including early drug discovery phases that target the identification of novel compounds along with physiological or disease modeling. While intestine, liver, and kidney OOCs can provide information on drug absorption, metabolism, and excretion profiles, pregnancy-related OOCs can provide insights into how the drug is distributed in both maternal and fetal compartments. Such information provides preliminary data on whether the drug would reach therapeutic dosage for the mother and the fetus, depending on who is being treated, and whether it reaches toxic levels in the fetal compartment (Kammala et al., 2023; Menon et al., 2024). Overall, the field is poised to make substantial changes in the area of pharmacology and provide stringent testing for therapeutics before phase trials. Time will only tell if existing OOC platforms will advance and overcome the current limitations of these systems to achieve their ultimate utility and purpose in the field of translational research.

## Future direction

As the reproductive OOC field moves forward devices will need to 1) be scaled down and “miniaturized” to create high throughput platforms, 2) integrate automatic cell/media loading to increase reproducibility and scalability, 3) incorporate patient-specific samples to create personalized medical approaches, 4) increase their biological complexity (incorporating immune cells, microbiomes, and linking multi-organ systems), 5) utilize physiologically relevant cutting edge endpoints, and 6) develop combinatorial novel approach methods such as artificial intelligence and machine learning to extrapolate and predict clinically relevant results from *in-vitro* experiments. Regardless of the methodology, OOC experiments should be detailed in a manner that allows for easy replication of results, thereby improving the reproducibility of findings and strengthening researchers, clinicians, and federal regulators’ trust in these platforms. STAR methods (Menke et al., 2020; Tonzani and Fiorani, 2021) should be incorporated into manuscripts utilizing OOCs and data generated from these devices should be deposited into Eva Analytics [MPS database (Gough et al., 2016)] to promote the wide-scale use of these devices as key tools to move the scientific field forward.
